# Validation of a lung ultrasound protocol in acute respiratory failure: preliminary results

**DOI:** 10.1186/cc12085

**Published:** 2013-03-19

**Authors:** RV Cremonese, AC Tabajara Raupp, JM Stormovski de Andrade, RS Townsend, FS Neres, AS Machado, MC Prestes, JG Maccari, FLDexheimer Neto

**Affiliations:** 1 Ernesto Dornelles, Porto Alegre, Brazil; 2 Hospital Moinhos de Vento, Porto Alegre, Brazil

## Introduction

Patients with acute respiratory failure demand dynamic evaluation and interventions. Lung ultrasound is a bedside technique, very promising in this setting. Previously, Lichtenstein and colleagues proposed an algorithm approach with a diagnostic accuracy of 90.5%, named Bedside Lung Ultrasound in Emergency (BLUE protocol). However, this approach has never been validated in other populations or ultrasound operators. The aim of this study was to evaluate the diagnostic accuracy of the lung ultrasound algorithm (BLUE protocol) in ICU patients admitted with acute respiratory failure.

## Methods

A prospective study, single-center consecutive case series evaluating critically ill adult patients in acute respiratory failure, admitted to a 23-bed general ICU between October 2011 and November 2012. The ultrasound examination was performed by an ICU physician until 20 minutes after admission, without interfering with usual care, and the ultrasound operator was blinded to the medical history of the patient. Three items were assessed: artifacts (horizontal A lines or vertical B lines indicating interstitial syndrome), lung sliding, alveolar consolidation and/or pleural effusion. BLUE protocol results were compared with clinical diagnosis by the medical assistant team at the end of the ICU stay.

## Results

Forty-two patients were evaluated (42% male), mean age was 73 years (SD ± 15) and APACHE II score was 18.77 (median 20). Based upon evaluation of the findings of BLUE protocol, a positive correlation was found in 83.78% of the sample. For the diagnosis of pulmonary edema (*n *= 15), sensitivity was 85% and specificity was 86%, while for the diagnosis of pneumonia (*n *= 17), sensitivity was 88% and specificity was 90%. In this sample there were only four cases of reversible airway obstruction and one case of pulmonary embolism. Five patients were excluded of the analysis because they had rare diagnoses.

## Conclusion

Preliminary results demonstrate diagnostic accuracy of lung ultrasound examination performed on the admission of patients with acute respiratory failure similar to the original publication. Lung ultrasound, as proposed in the BLUE protocol, has good accuracy, and seems reproducible and useful in this group of patients.
